# Prevalence and risk factors for intestinal parasitic infections in pregnant women residing in three districts of Bogotá, Colombia

**DOI:** 10.1186/s12889-018-5978-4

**Published:** 2018-08-29

**Authors:** Angela Fernanda Espinosa Aranzales, Katja Radon, Guenter Froeschl, Ángela María Pinzón Rondón, Maria Delius

**Affiliations:** 1 Escuela de Medicina y Ciencias de la Salud, Universidad del Rosario, Bogotá, Colombia; 2 Center for International Health, Medical Center of the University of Munich (LMU), Munich, Germany; 3 Institute and Outpatient Clinic for Occupational, Social and Environmental Medicine, University Hospital of Munich (LMU), Munich, Germany; 4 Division of Infectious Diseases and Tropical Medicine, Medical Center of the University of Munich (LMU), Munich, Germany; 5 Department of Obstetrics and Gynecology, University Hospital, LMU Munich, Munich, Germany

**Keywords:** Intestinal parasitic infections, Pregnant women, Vulnerable populations, Sociodemographic factors, Housing, Living conditions

## Abstract

**Background:**

Intestinal parasitic infections (IPI) lead to significant morbidity and mortality in pediatric and adult populations worldwide. Intestinal parasitism during pregnancy is of interest as it may affect the health of pregnant women and their offspring. This study determined the prevalence of IPI in pregnant women living in substandard conditions in three urban districts of Bogotá, Colombia. Associations between prevalence and sociodemographic factors, housing, and living conditions were also evaluated.

**Methods:**

In a cross-sectional and community-based study, pregnant women were recruited from three districts of Bogotá. A total of 550 participants answered a questionnaire; 331 of these also provided stool samples, with 233 providing one and 98 providing two stool samples. Questionnaire responses were associated with the presence of intestinal parasites, which was determined using a standard combined microscopy technique including direct wet mount and formol–ether concentration. Results were verified by supplementary examination of 48 stool samples by quantitative polymerase chain reaction (qPCR).

**Results:**

Among pregnant women who lived in selected poor residential areas in Bogotá, the overall prevalence of intestinal parasitism was 41% with 9% polyparasitism. Pathogenic parasites were present in 1.2% of the 331 participants including *Giardia lamblia* and *Ascaris lumbricoides*. Higher prevalence was found for parasites with debated pathogenicity, including *Blastocystis hominis* (25%), *Endolimax nana* (15%), *Entamoeba coli* (8%), and *Iodamoeba butschlii* (2%). *Entamoeba histolytica/dispar* complex was also detected (1.5%). When comparing a subset of stool samples using the combined microscopy technique and qPCR, the latter detected a higher 58.3% overall IPI prevalence. Higher prevalence of infections by any intestinal parasite was found in participants who had never been dewormed (*p* = 0.01). Higher but not statistically significant associations were found between any parasite and women living with a partner, and intestinal polyparasitism and being from a minority group and not having a water sink.

**Conclusions:**

This first study of the prevalence of intestinal parasitism in Bogotá focused on pregnant women living in poverty, found a high prevalence of intestinal parasites of debated pathogenicity, and confirmed a low prevalence of pathogenic intestinal parasites. These results highlight the need for educational interventions to disrupt transmission routes for prevalent parasites.

## Background

The World Health Organization (WHO) estimates that at least one quarter of the world’s population is infected with soil-transmitted helminths [1]. Consequences of infections are compromised growth, cognitive impairment, malnutrition, and anemia [2–4]. Worldwide, about 300 million people suffer from severe helminth infections, leading to morbidity and over 150,000 deaths annually [5]. Amoebiasis caused by *Entamoeba histolytica* kills between 40,000 and 100,000 people per year [6], whereas giardiasis is the main cause of parasitic diarrhea worldwide [7] and an important cause of waterborne disease outbreaks [8, 9]. Housing conditions are an important determinant for developing intestinal parasitic infections (IPI) [4, 10, 11]. Risk factors for these infections include deprivation of access to clean water, inadequate hygiene habits, and inferior sanitary conditions [12–14]. As in the case of other neglected diseases, poverty in general is a condition correlating with IPI [15]. Although factors associated with parasitism in pregnant women are mainly the same as in other population groups [16], multiparity is an additional risk factor [17]. On a larger scale, people living below the poverty line in low-income countries, especially young women and young pregnant women, their infants, and children, are at a high risk of IPI [2]. Among these high-risk groups, IPI studies have focused primarily on children, while data on women of childbearing age are scarce. Therefore, considering that IPI have significant consequences for pregnant women and their offspring, including maternal anemia [4, 18], low pregnancy weight gain, poor fetal growth [19], low birth weight [20], and preterm birth [4], pregnant women are an important population for study. Among intestinal parasites, the WHO considers soil-transmitted helminthiasis (STH) as the most common infections in vulnerable populations [5]. Prevalence studies in Latin American populations report wide ranges from 1% up to 97% [2]. In Colombia, STH prevalence data range between 11 and 50% [2, 21]. More specifically, for Bogotá, the capital city, prevalence has been reported to be from 1.5 to 10% [22].

The most common enteric protozoa in humans are *Giardia lamblia*, *Entamoeba histolytica*, and *Blastocystis hominis* [23, 24]. Although giardiasis has a ubiquitous distribution, it is more prevalent in developing countries [25]. Its worldwide prevalence of about 3% [26] contrasts with 8–67% reported for Latin America as a whole [24, 27, 28], 13–17% for Colombia [21, 29], and 12–20% for Bogotá [22, 30, 31]. *Entamoeba histolytica* infection, estimated to affect 12% of the population worldwide [32], is more prevalent in tropical regions. In Latin America, prevalence has been reported between 4 and 12% [19, 33, 34], in Colombia between 0 and 54% [21, 29, 35, 36], and in Bogotá between 0 and 3% [22, 30]. With its pathogenicity still debated [12], worldwide prevalence of *Blastocystis hominis* has been reported to be as high as 100% [37, 38], while in Latin America it is reported to be between 22 and 67%, in Colombia between 6 and 54% [21, 29, 39], and at 3% in Bogotá [30].

Bogotá lies in the center of Colombia at 2600 m above sea level (4°35′56 N, 74°04′51 W) and is located within an intertropical zone with an annual average temperature of 14 °C and about 80% humidity [40]. Despite being the largest and most developed city in Colombia, Bogotá exhibits great inequality between rich and poor, reflected in a 2016 GINI index of 0.499 [41]. Of its 8 million inhabitants [42], 9.2% of the population of Bogotá cannot afford one or more of their basic needs, as defined by the Colombian National Administrative Department of Statistics [43], 11.6% live in poverty, and 2.3% live in extreme poverty. It is estimated that more than 400,000 displaced people live in Bogotá [44], most of them living in conditions of inadequate sanitation and overcrowding [45]. Bogotá has an urban area of 384 km^2^ [46] divided into 20 districts, which are administrative units with different features and resources and, just as in the rest of the country, are categorized into socioeconomic strata based on housing and neighborhood conditions. Strata range from 1, indicating substandard housing conditions, to 6 with high-level housing conditions [47]. Stratification is based on a reference index of living standards, inequality, and poverty. Geographically, strata identify regions in which people share similar social and economic characteristics [48]. A given urban district may contain several different strata.

Following the Colombian Ministry of Health and Social Protection, adhering to the 2002 WHO recommendations, Colombia has implemented preventive antiparasitic treatments in at-risk populations [49]. According to these guidelines, control programs target mostly healthy school-aged children who live in endemic areas by providing prophylactic and periodical broad-range antihelminthic treatments, with suboptimal coverage below the 75% target. In large non-endemic urban areas such as Bogotá, children should receive one yearly prophylactic dose, but pregnant women should not receive these prophylactic treatments. In parallel, community-based control programs focus on education, hygiene habits, food handling, and adequate public and residential sanitation services to disrupt parasitic life cycles.

This study investigated the magnitude of IPI in pregnant women in poor residential areas in Bogotá and identified risk factors associated with these infections. The results and recommendations of this study will provide evidence for stakeholders in health care and public health, geared toward implementing and improving preventive measures in pregnant women, especially in poor residential areas.

## Methods

### Study design

**Fig. 1:**
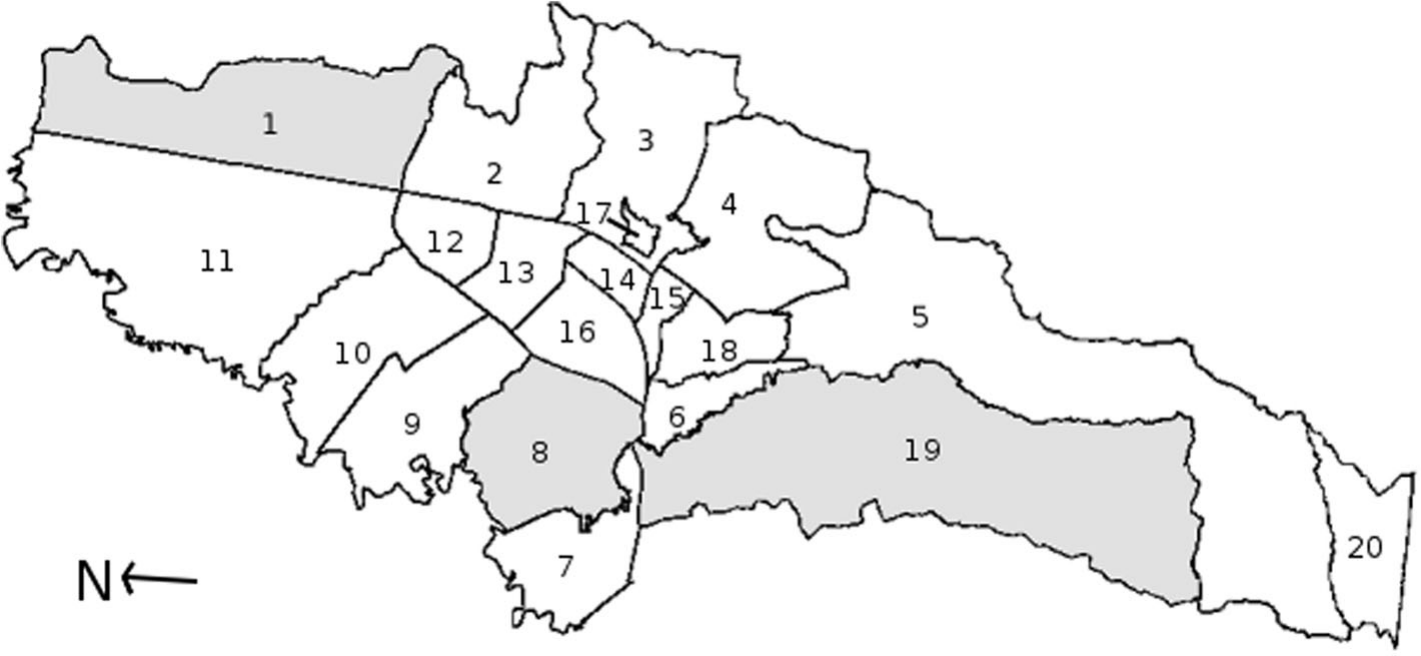
Map of Bogotá districts. Three districts were selected to study the prevalence of intestinal parasitism in pregnant women living in the largest city in Colombia. 1. Usaquen District. 8. Kennedy District. 19. Ciudad Bolivar District. Adapted from: Louise Wolff, 2006 (Own work, Public Domain; commons license permission granted worldwide to use for any purpose and without any conditions) [92]

A cross-sectional, community-based study, in which participants answered a questionnaire and provided one or two stool samples, was conducted between May 2015 and July 2016 in Bogotá. This study focused on pregnant women living in poor residential areas in three districts of Bogotá (Fig. 1). These communities were selected because they have a majority of strata 1 and 2 neighborhoods or receive a large number of people displaced within Colombia [50], who live in socioeconomic and geographical conditions that may affect their risk of IPI. The selected districts were Usaquén (population 472,908; area 65.31 km^2^), Kennedy (population 1,187,315; area 38.56 km^2^), and Ciudad Bolívar (population 719,700; area 129.98 km^2^) [42, 51].

### Study population and sample

Colombia’s health care system is organized into three levels of attention with primary care units (Unidad Primaria de Atención or UPA) within a basic level of health care and secondary and tertiary care hospitals. In Bogotá, antenatal care in public health settings is provided in UPAs with high-risk cases being referred. The study’s target population was all pregnant women in any trimester, living in the districts of Usaquén, Kennedy, and Ciudad Bolívar, belonging to strata 1 or 2, and attending antenatal care in local primary care units. During the study period, a total of about 9600 pregnant women attended these units.

To calculate the sample size in this study, the Epi Info™ 7.2 software developed by the Center for Disease Control and Prevention (CDC, Atlanta, GA, USA) was used. More specifically, using the StatCalc module for cross-sectional studies, sample size was estimated based on exposure and outcome prevalence reported in previous similar studies in Colombian and Venezuelan cohorts. These reported 43%, 12%, and 9.5% parasitism in groups exposed to unfavorable socioeconomic and sanitary conditions, in contrast to 33% and estimated 6% and 4.5% in unexposed groups [22, 24], with estimated ORs of 1.53, 2.14, and 2.23 respectively. The different sample sizes were determined with a level of accuracy of 5% and statistical power of 80%. Thus, sample sizes of 776, 778, and 894 were calculated. Considering a non-response proportion of 20%, the resulting estimated sample size was 1100 pregnant women.

### Data collection

With respect to national data protection regulations, it was not possible to access patient databases directly to establish a sample frame of pregnant women who attended the primary care units within the three districts. Therefore, from May 2015 to July 2016, research assistants proactively invited pregnant women to participate in the study. Fieldwork was supported by six research assistants experienced in community work who had received training in technical, ethical, and logistic procedures before data collection. Once the informed consent form was signed, each woman received a kit and instructions for appropriate stool collection. Sociodemographic aspects and pregnancy characteristics were assessed at the UPAs through individual interviews, while data related to housing conditions and hygiene habits were assessed through questioning complemented with inspection during home visits. Once the questionnaire was completed, pregnant women received an educational intervention about general health recommendations and danger signs during pregnancy. Afterwards, participants received their exam results with a recommendation to include them in the next visit to the physician. Fieldwork started in the districts of Usaquén and Kennedy. In these districts, research assistants approached pregnant women directly before and after prenatal courses at UPAs and, in addition, in Usaquén, they included door-to-door visits. Ciudad Bolívar was the last district, in which research assistants systematically invited every pregnant woman who attended any time between 7 am and 4 pm at two UPAs selected within the district because both received 80% of pregnant women from the district. In the district of Usaquén, recruitment had to be suspended as a result of unsafe working conditions for research assistants when neighborhood conditions deteriorated during the period of fieldwork. This led to an underrepresented 4% (24/550) sample of participants in this district. In the district of Kennedy, the recruitment of participants was stopped because of selective sampling in favor of the pregnant women who could attend courses at the UPA. Consequently, only 23% (127/550) of the study participants were from that district. For these reasons, 73% of the participants were from Ciudad Bolívar, the district of Bogotá where 59% of people live in stratum 1 and 38% in stratum 2 [52].

Of the 210 participants who did not provide stool samples, 95% (199/210) were from the district of Ciudad Bolívar, of whom 84% (167/199) resided in stratum 1 (Table 1). This district is located on a mountain with the poorest homes near the top, where access is difficult and not always feasible for geographical and safety reasons. Despite these limitations in accessing these participants, research assistants attempted data collection when conditions allowed. Additionally, some pregnant women who initially answered the questionnaire in the local primary care units did not accept the home visit to pick up the stool sample and did not hand in the sample to the units.

**Table 1:** Characteristics of participants who answered the questionnaire and participants who also provided one stool sample

Characteristic	Participants with questionnaires only			Participants with questionnaires and stool samples			*p* value
	Total: 210			Total: 340			
	N_missing_	N	%	N_missing_	N	%	
City district of Bogotá							
Ciudad Bolívar	0	199	94.8	0	200	58.8	< 0.001
Kennedy		9	4.3		118	34.7	
Usaquén		2	1.0		22	6.5	
Stratum^a^							
One	0	168	80.0	0	172	50.6	< 0.001
Two		42	20.0		168	49.4	
Ethnicity							
Minority group^b^	0	14	6.7	1	24	7.1	0.5
Majority group		196	93.3		315	92.9	
Occupation							
Family maker/housewife	0	156	74.3	0	265	77.9	0.436
Student		19	9.0		35	10.3	
Sales and services		19	9.0		22	6.5	
Other		16	7.6		18	5.3	
Level of education							
Elementary school	0	35	16.7	0	38	11.2	0.116
Secondary school		147	70.0		243	71.5	
Higher education		28	13.3		59	17.4	
Civil status							
Single	0	67	31.9	0	103	30.3	0.380
Married or cohabiting		143	68.1		237	69.7	
Covered by social health insurance							
Yes	0	168	80.0	0	300	88.2	0.010
No		42	20.0		40	11.8	
Victim of forced displacement							
Yes	0	41	19.5	0	66	19.4	0.974
No		169	80.5		274	80.6	
Monthly income^c^							
≤ 1 Minimum wage	48	108	66.7	18	245	76.1	0.088
> 1 Minimum wage		54	33.3		77	23.9	
Parity							
Nulliparous	2	100	48.1	1	185	54.6	0.140
Multiparous		108	51.9		154	45.4	
Trimester							
First	13	43	21.8	6	63	18.9	0.202
Second		74	37.6		152	45.5	
Third		80	40.6		119	35.6	
Last deworming of participant							
Less than 1 year ago	31	28	15.7	32	62	20.1	0.200
More than 1 year ago		77	43.0		109	35.4	
Never		74	41.3		137	44.5	

^a^Socioeconomic classification in Colombia

^b^Afrocolombian and native ethnic people

^c^Minimum monthly Colombian income (for 2016) = USD 233

A total of 775 pregnant women were invited to participate: of these, 71% (550/775) accepted the invitation; of the latter, 94% (519/550) answered the questionnaire completely and 62% (340/550) gave one stool sample. Of the 340 participants who provided a stool sample, 32% (107/340) provided a second stool sample, nine of which had to be excluded because of processing errors. Thus, this study included 331 participants with at least one stool sample, 98 of whom provided a second stool sample. In addition, of the 331 participants, a subgroup of 50 samples was processed further for quantitative polymerase chain reaction (qPCR) investigation. Two of these samples were lacking formol–ether concentration data, so they were excluded from further comparison, resulting in 48 samples included for molecular analysis.

### Questionnaire

The interviewer-administered questionnaire was adopted from the Demographic and Health Surveys (DHS) to assess sociodemographic conditions as implemented by ICF International (Fairfax, VA, USA) [53]. The items included were age, occupation, education level, civil status, health insurance coverage, monthly income, household conditions, water availability, supply, and sewage, and hygiene habits such as boiling water before drinking, washing fruit and vegetables, place and reasons for washing hands throughout the day, and habits of walking barefoot. Two research members (AE and KR) translated the questionnaire from English into Spanish. In addition, questions about parity and trimester of pregnancy were taken from a Spanish form on “Pregnant women in primary health care” [54]. Items on garbage collection, vectors, and living with pets were chosen from the Spanish form on “Elementary family characteristics” [55], both developed by the Health District Secretary of Bogotá. Supplementary questions regarding the location of the house, socioeconomic stratum, and date of the last deworming procedure were included. A pilot test of the questionnaire to control for clarity, comprehension, and duration of the survey was carried out with three students from Universidad del Rosario, Bogotá. They were in their fifth year of medical school and received training by two investigators (AE and AP) to apply the survey. Ten women residing in the district of Usaquén who were in their last month of pregnancy were interviewed once they gave their informed consent. These women were not included as participants in the study. Minor wording adjustments were made following their feedback.

### Stool sample collection and laboratory methods

All study participants received stool sample containers and standard instructions on proper and safe collection and preservation of the samples. By agreement, research assistants contacted participants by phone to insure that, once the sample was collected and stored in proper conditions (inside the fridge or in a dark low-temperature home location), the assistants could pick it up from the participants’ homes no more than 4 h after evacuation. The research assistants, who were trained in biosafety standards, followed a protocol for stool sample collection and handling, including appropriate labeling, triple packaging [56], keeping the cooling chain, and ensuring proper, timely, and safe handling and delivery of stool samples to the laboratory.

### Combined microscopy technique

The detection techniques selected in this study were based on diagnostic performance, methodological availability, feasibility, and cost-effectiveness [57]. In this study, each stool sample was analyzed by a so-called “combined microscopy technique”, which included sample analysis by direct wet mount microscopy and by a formol–ether concentration method and subsequent microscopy [58]. Both methods were performed by a contracted specialized nationwide reference laboratory following standard procedures. Direct wet mount microscopy has reportedly shown better diagnostic capacity for protozoal trophozoites, especially *Giardia* [57] and *Blastocystis* [59], while the concentration technique has demonstrated better performance for other protozoa [59] and for helminths [60, 61].

### qPCR

In this study, a subgroup of 48 stool samples was subsequently analyzed by qPCR for comparison with the results obtained by standard microscopy detection techniques. These samples originated from participants residing in Ciudad Bolívar district and were selected prospectively and chronologically by order of arrival at the laboratory. For financial reasons, not all samples could be analyzed by qPCR. The qPCR technique, with 100% primer-determined specificity and reported sensitivity close to 100% [62, 63], was carried out by the microbiology laboratory of Universidad del Rosario, Bogotá.

At the university laboratory, the stool sample was fixed with 70% ethanol. With 300 μl of the fixed sample, DNA was extracted using the Norgen Stool DNA isolation kit (Norgen Biotek Corporation, Thorold, Canada) following the manufacturer’s protocol. The PCR was conducted on a total volume of 7 μl containing 3.5 μl of Taqman Mastermix™ (Applied Biosystems, Foster City, CA, USA), 2 μl of DNA, and 1.5 μl of species-specific primers (final concentration = 900 mM) and Taqman probes (final concentration = 100 mM). Samples were run in an Applied Biosystems 7500 Fast Real-Time PCR system and processed using a denaturation time of 3 s at 95 °C and an extension time of 30 s at 60 °C for 40 cycles. The results of qPCR were considered negative if the cycle threshold values (Ct) were > 38. This threshold was determined by measuring the detection limits of each assay in which serial dilutions of parasites were included [64]. The available PCR primers were: *Blastocystis hominis*, *Giardia lamblia*, *Cryptosporidium*, *Entamoeba histolytica*, *Ancylostoma duodenalis*, *Necator americanus*, *Ascaris lumbricoides*, and *Trichuris trichiura*, all of which were based on the primers reported by Mejia et al. [64], except the primer for *B. hominis*, which was based on Stensvold et al. [65]. A reference strain of *Giardia duodenalis* WB and DNA from each of the parasites were used as positive controls. Stool samples previously collected and confirmed by the laboratory to be from healthy non-infested children served as negative controls.

### Data analysis

All data were double entered and controlled for errors. Data analysis was performed using IBM SPSS Statistics version 24 software (Armonk, NY, USA). A comparison of sociodemographic characteristics was performed between the 210 pregnant women who answered the questionnaire only and the 340 participants who also provided a stool sample.

Laboratory reports indicated the presence of any parasite form (trophozoites, cysts, eggs, or larvae) as detected by direct and concentration techniques separately and for each parasite. With these data, one variable for each parasite was defined as “negative = 0” (if no form was reported) or “positive = 1” (if any parasite form was reported). Once the prevalence of each parasite was established with each technique, a pooled analysis with both techniques was generated. A combined variable for each parasite was created and defined as “negative = 0” (if no parasite was detected by either technique) or as “positive = 1” (if any parasite was detected by either technique). In all included participants, the prevalence was analyzed in three dimensions, namely the presence of any parasite, the presence of any pathogenic parasite, and the presence of more than one parasite (“polyparasitism”). For each outcome dimension, one compound variable was created and defined as “negative = 0” (if no parasite forms were detected with the combined microscopy technique) or “positive = 1” (if parasite forms were detected with the combined microscopy technique).

When qPCR was added to the combined microscopy technique in a subgroup of participants, the prevalence of each parasite was established separately and combined by creating variables, as described above. The percentage of agreement between the two techniques was then determined. Positive agreement percentage was estimated as a proportion corresponding to the number of parasites detected by combined microscopy technique and confirmed by qPCR over the total number of parasites detected by qPCR. Negative agreement was estimated as a proportion corresponding to the number of stool samples reported as negative for parasites by combined microscopy technique and confirmed by qPCR over the total negative cases detected by qPCR [66].

Exposure variables such as sociodemographic and pregnancy characteristics, living conditions, and hygiene habits were evaluated as categorical variables and presented as absolute and relative frequencies. Age was evaluated numerically. Bivariate analysis was done using Pearson’s Chi-square (χ^2^) test, Fisher’s exact test, or Mann–Whitney test as appropriate. Factors that correlated highly with the polyparasitism variable (*p* < 0.1) were included in a logistic regression analysis.

## Results

### General characteristics of the participants

Of the 550 pregnant women who agreed to participate, 38% (210/550) only answered the questionnaire and 62% (340/550) responded to the questionnaire and handed in one stool sample. Sociodemographic conditions based on participant characteristics were compared between participants only responding to the questionnaire and those additionally providing a stool sample. Statistically significant differences were identified according to district, stratum, and health insurance coverage between participants who only answered the questionnaire and those who also provided a stool sample (*p* < 0.001). Among the women who answered the questionnaire only, 94.8% (199/210) resided in Ciudad Bolívar, 80% (168/210) lived in stratum 1, and 80% (168/210) were covered by social health insurance (Table 1). In addition, the median age for participants who only answered the questionnaire was 21 years (range 15–41 years), while the median age for those who also provided a stool sample was 22 years (range 14–43 years), *p* = 0.034. All other variables showed no statistically significant differences.

### Prevalence of intestinal parasitic infections in pregnant women

**Fig. 2:**
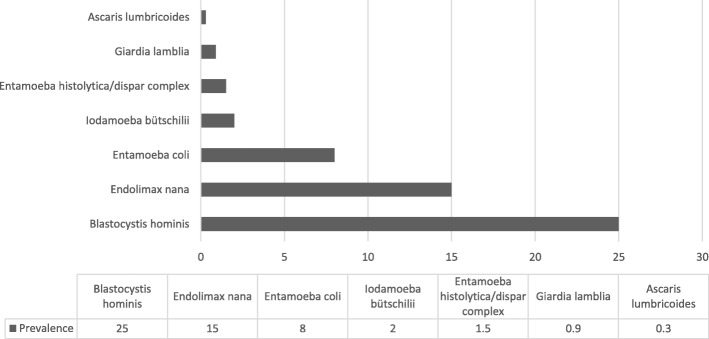
Prevalence of intestinal parasitic infections in the 331 pregnant women living in poor residential areas of Bogotá. Seven intestinal parasites were found in the stool samples of participants using a combined microscopy technique

Nine participants from the 340 (3%) who delivered at least one stool sample had to be excluded because of processing errors in the laboratory. Of the remaining 331 participants, 107 (32%) voluntarily provided a second stool sample, and nine of the 107 (8%) had to be excluded because of processing errors in the laboratory. The 331 participants with at least one analyzed stool sample will serve as denominator in the following analyses. Among those who provided one stool sample, 41% (CI 95% 35.7–46.3) (137/331) had at least one intestinal parasite, either pathogenic or non-pathogenic, and 9% (CI 95% 5.9–12.0) (31/331) had more than one intestinal parasite (polyparasitism). The overall prevalence of any pathogenic parasites was 1.2% (CI 95% 0.0–2.4) (4/331), consisting of two parasite species. As shown in Fig. 2, the prevalence of *Giardia lamblia* was 0.9% (3/331) and of *Ascaris lumbricoides* was 0.3% (1/331). The findings for the *Entamoeba histolytica/dispar* complex have to be regarded separately, as this parasite was only detected in those samples that were investigated by microscopy-based methods; hence, a differentiation between *Entamoeba histolytica* as a pathogenic species and *Entamoeba dispar* as a non-pathogenic species, which is only possible by nucleic amplification techniques, was not available. The prevalence of the complex was 1.5% (5/331). Regarding non-pathogenic parasites, the prevalence was 25% (83/331) for *Blastocystis hominis*, 15% (50/331) for *Endolimax nana*, 8% (26/331) for *Entamoeba coli*, and 2% (6/331) for *Iodamoeba butschlii*.

Of the 98 participants who provided a second stool sample, when both the first and second samples were analyzed together with combined microscopy techniques, the prevalence of any parasite was 52% (51/98), with 14% (14/98) for more than one parasite. The only pathogenic intestinal parasite detected was *Giardia lamblia* in 3% of the samples (3/98). For non-pathogenic parasites, the prevalence of *Blastocystis hominis* was 36% (35/98), of *Endolimax nana* was 22% (22/98), of *Entamoeba coli* was 9% (9/98), and of *Iodamoeba butschlii* was 3% (3/98). In addition, the prevalence of *Entamoeba histolytica/dispar* complex was 2% (2/98).

**Fig. 3:**
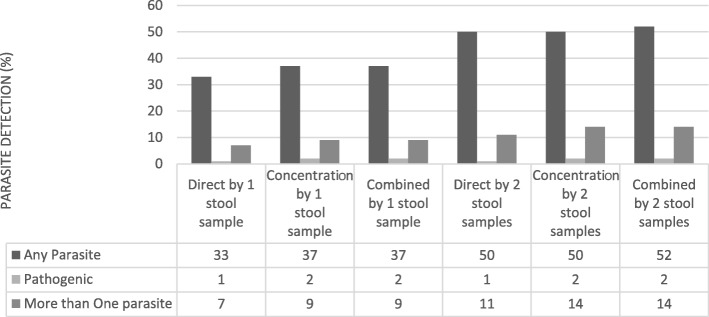
Parasite detection by three methods with one and two samples (*n* = 98). Detection of any parasite and polyparasitism is increased with a combined microscopy technique and with two samples

As seen in Fig. 3, the second stool sample increased detection from the first to the second stool sample from 37% (33/98) to 52% (51/98) for any parasite and from 9% (9/98) to 14% (14/98) for polyparasitism, while it remained unchanged for pathogenic parasites at 2% (2/98).

### Comparison of qPCR with the combined microscopy technique

Test results for a subset of 48 samples were compared between qPCR results for eight selected parasites, as outlined in the methods section, and the combined microscopy-based techniques. Two of these 50 samples could not be conclusively processed in the combined microscopy technique and therefore had to be excluded from this sub-analysis. For the eight investigated parasites, qPCR identified only two types of parasites, *B. hominis* in 54% (26/48) and *G. lamblia* in 4% (2/48). The combined microscopy technique identified *B. hominis* in 27% (13/48) and 0% (0/48) for *G. lamblia*. When comparing both techniques to identify the presence of any parasite in the samples, the prevalence estimated by qPCR was 54% (26/48) in contrast to 31% (15/48) with the combined microscopy technique (*p* Fisher < 0.004). The positive and negative agreements to diagnose any parasite were 50% (13/26) and 91% (20/22), respectively, while positive and negative agreements to diagnose *B. hominis* were 48% (12/25) and 96% (22/23) respectively. With *G. lamblia*, there was a positive agreement of 0% and a negative agreement of 96% (Table 2).

**Table 2:** Comparison between qPCR test and a combined microscopy technique in a subset of 48 participants

	Prevalence by combined microscopy technique % (n)	Prevalence by qPCR technique % (n)	Positive agreement %^b^ 95% CI	Negative agreement %^c^ 95%CI
*B. hominis*	27.0 (13)	52.0 (25)	48.0 30.0–66.5	95.7 79.0–99.2
*G. lamblia*	0.0 (0)	4.0(2)	0.0 0.0–0.0	95.8 90.2–100
Any parasite^a^	31.0 (15)	54.0 (26)	50.0 32.1–67.9	90.9 72.2–97.5

*n* number of positive study participants diagnosed by each individual test

% = percentage

*CI* confidence interval

^a^“Any parasite” means that, in the sample analyzed, at least one parasite has been identified, regardless of pathogenicity

^b^Positive agreement percentage corresponds to the number of parasites detected by combined microscopy technique and confirmed by qPCR, over the total number of parasites detected by qPCR

^c^Negative agreement percentage corresponds to the number of stool samples reported as negative for parasites by combined microscopy technique and confirmed by qPCR, over the total negative cases detected by qPCR

### Factors associated with infection by any parasite and intestinal polyparasitism

Associations of infection by any parasite and intestinal polyparasitism with sociodemographic characteristics (Table 3), living conditions, and hygiene behaviors (Table 4) were tested. A higher significant association was found between infection by any parasite and last deworming: less than 1 year ago with 34% (20/59) prevalence, more than 1 year ago with 52% (55/106) prevalence, and never with 36% prevalence (48/135) (*p* = 0.01). For infection by any parasite, a higher but non-significant association was found with civil status: being single, 34% (34/101) prevalence; and living with a partner, 45% (103/230) prevalence (*p* = 0.06). There was no statistically significant difference for the variable of age between women with any parasite infection (median age 21 years (range 14–43 years)) and women without any parasite infection (median age 23 years (range 14–40 years)) (*p* = 0.16). Higher but non-significant associations with polyparasitism were found for women from minority groups with 21% (5/24) prevalence compared with 9% (26/306) for women from majority groups (*p* = 0.06), and for women without a water sink in the toilet with 14% (14/100) prevalence compared with 7% (17/230) for those possessing a water sink (*p* = 0.06). There was no statistically significant difference for the variable of age between women with polyparasitism (median age 22 years (range 17–37 years)) and women without polyparasitism (median age 22 years (range 14–33 years)) (*p* = 0.09). Similarly, there were no significant differences when polyparasitism was compared between parous women at 12% (18/149) and nulliparous women at 7% (13/181) (*p* = 0.09). A multivariate analysis was performed including variables in which associations in bivariate analysis resulted in *p* values lower than 0.10. For infection by any parasite, the analyzed variables were civil status and last deworming. The only variable that remained in the equation was last deworming (more than 1 year *p* = 0.82, OR 0.929, CI 0.488–1.770; never dewormed *p* = 0.011, OR 1.955, CI 1.163–3.284). For polyparasitism, the analyzed variables were ethnicity, place to wash hands, age, and parity. The only variable that remained in the equation was ethnicity (*p* = 0.057, OR 2.82, CI 0.971–8.154).

**Table 3:** Prevalence of any parasite infection and intestinal polyparasitism by sociodemographic characteristics of 331 participants

Characteristic	N	Any parasite			Polyparasitism		
		n	%	*p* value	N	%	*p* value
City district of Bogotá							
Ciudad Bolívar	194	87	44.8	0.11	19	9.8	0.73
Kennedy	115	45	39.1		11	9.6	
Usaquén	22	5	22.7		1	4.5	
Stratum^a^							
One	168	74	44.0	0.32	14	8.6	0.64
Two	163	63	38.7		17	10.1	
Ethnicity							
Minority group^b^	306	124	40.5	0.37	26	8.5	0.06
Majority group	24	12	50.0		5	20.8	
Occupation							
Housewife	259	105	40.5	0.38	27	10.4	0.50
Student	33	13	39.4		1	3.0	
Sales and services	21	8	38.1		1	4.8	
Other	18	11	61.1		2	11.1	
Level of education							
Elementary school	38	20	52.6	0.30	3	8.0	0.93
Secondary school	234	92	39.3		22	9.4	
Higher education	59	25	42.4		6	10.2	
Civil status							
Single	101	34	33.7	0.06	10	9.9	0.82
Married or cohabiting	230	103	44.8		21	9.1	
Social health security coverage							
Yes	291	120	41.2	0.88	25	8.6	0.19
No	40	17	42.5		6	15.0	
Victim of forced displacement							
Yes	267	107	40.1	0.32	23	8.6	0.34
No	64	30	46.9		8	12.5	
Monthly income^c^							
≤ 1 Minimum wage	73	29	39.8	0.99	6	8.2	0.81
> 1 Minimum wage	241	96	39.7		22	9.1	
Parity							
Nulliparous	181	76	42.0	0.75	13	7.2	0.13
Parous	149	60	40.3		18	12.1	
Trimester							
First	61	21	34.4	0.46	7	11.5	0.50
Second	149	64	43.0		16	10.7	
Third	115	50	43.5		8	7.0	

The following variables had missing data: ethnicity (1 missing), monthly income (17 missing), parity (1 missing), and trimester (6 missing)

^a^Socioeconomic classification in Colombia

^b^Afrocolombian, native ethnic people

^c^One minimum monthly Colombian income (for 2016 year) = USD 233

**Table 4:** Prevalence of intestinal parasitic infections by living conditions and hygiene habits of 331 participants

Characteristic	N	Any parasite			Polyparasitism		
		n	%	*p* value	N	%	*p* value
Piped water supply							
Yes	314	129	41.1	0.63	29	9.2	0.66
No	17	8	47.1		2	11.8	
Sewage							
Yes	315	129	41.0	0.47	29	9.2	0.65
No	16	8	50.0		2	12.5	
Garbage collection							
> 2 times per week	277	112	40.4	0.44	26	9.4	0.96
≤ 2 times per week	52	24	46.2		5	9.6	
Presence of pets							
No	135	55	40.7	0.84	11	8.1	0.52
Yes	196	82	41.8		20	10.2	
Last deworming of participant							
Less than 1 year ago	59	20	33.9	0.01	5	8.5	0.32
More than 1 year ago	106	55	51.9		13	12.3	
Never	135	48	35.6		9	6.7	
Boiling water before drinking							
Yes	122	51	41.8	0.89	12	9.8	0.84
No	207	85	41.1		19	9.2	
Washing fruit and vegetables							
Yes	317	131	41.3	0.90	30	9.5	1.00
No	14	6	42.9		6	15.0	
Place for washing hands at home							
Sink	230	91	39.6	0.28	17	7.4	0.06
Other^a^	100	46	46.0		14	14.0	
Water availability for washing hands at home							
From tap water	274	114	41.6	0.94	26	9.5	0.90
From water tank	56	23	41.1		5	8.9	
Washing hands before eating							
Yes	211	92	43.6	0.27	21	10.0	0.63
No	120	45	37.5		10	8.3	
Washing hands after going to the toilet							
Yes	280	112	40.0	0.23	27	9.6	0.69
No	51	25	49.0		4	7.8	
Walking barefoot at home							
No	177	73	41.2	0.95	14	7.9	0.33
Yes	154	64	41.6		17	11.0	

The following variables had missing data: garbage collection (2 missing), last deworming of participants (31 missing), boiling water before drinking (2 missing), place for washing hands at home (1 missing), water availability for washing hands at home (1 missing)

^a^Kitchen sink or scullery

## Discussion

This study estimated the prevalence of intestinal parasitism and associated environmental factors in pregnant women living in poor residential areas within three districts of Bogotá, Colombia. We found a 41% prevalence of pathogenic or non-pathogenic intestinal parasites in stool samples using combined microscopy-based testing. Intestinal infections by any parasite were significantly higher in pregnant women who had never been dewormed and higher, but not significantly so, in those who were married or cohabiting. Polyparasitism in women from minority groups and those not having a water sink in the bathroom showed an important but non-significant increase in prevalence.

Participant selection was challenging as recruitment by phone was not allowed on account of data protection laws. Also, at UPAs, scheduled control visits for pregnant women were mixed with all other medical appointments. Given these limitations, and as UPA staff suggested that pregnant women primarily attended psychoprophylaxis courses, it was decided to focus recruitment on these activities. Once this fieldwork started, preliminary analysis revealed that attendance at these sessions mostly included nulliparous women and housewives. Based on this, and the deteriorating safety conditions in Usaquén, fieldwork in Kennedy and Usaquén districts was suspended. As Ciudad Bolívar included a majority of residents living in strata 1 and 2, and two UPAs receiving up to 80% of pregnant women in the district, systematic recruitment was concentrated at this location. As this study aimed to identify environmental factors associated with prevalence, systematic data collection was favored over representative sampling, as stated by Rothman et al. [67]. In addition to the ethical obligation to protect the safety of research assistants, community leaders supported the recruitment and data collection processes as much as possible. Thus, safety and the recruitment limitations mentioned above, as well as the unexpectedly low prevalence of pathogenic parasites in the study cohort, did not justify intensifying the recruitment activities in order to reach the calculated targeted sample size. For these reasons, data collection was stopped after the recruitment of the 550 participants in this study.

Of the 775 women who were invited to participate, 26% (200/775) did not accept. This value was close to the 20% estimated non-response proportion initially predicted, congruent with empirical observations in community-based studies in Latin America. In 6% (31/550) of participants, house visits to observe hygiene practices and living conditions were not performed, as participants provided an incorrect address or did not attend the scheduled appointment.

Increased detection and prevalence were achieved with two stool samples, a finding congruent with Cartwright [68]. However, this was only ascertained with a subgroup of 107 participants who voluntarily provided a second stool sample. Double sampling could have been restricted by physiologic constipation inherent to pregnancy, for safety reasons, and limited geographical access. This increased detection confirmed the low prevalence of pathogenic intestinal parasites. In this study, the overall response rate for one stool sample was 60% (340/550), a rate considered challenging given the conditions mentioned above. Comparing pregnant women who only answered the questionnaire with those who also provided stool samples showed statistically significant differences by districts, socioeconomic strata, and health coverage. These variances related to recruitment drawbacks mainly caused by difficulties in fieldwork due to serious safety issues and the availability of pregnant women.

Although qPCR detected a higher prevalence of *Blastocystis* and *Giardia* than the combined microscopy technique, it confirmed the overall low prevalence of pathogenic parasites in this study. The low proportional positive agreement between the two techniques is expected, as qPCR is a technique with a sensitivity in stool samples close to 100% with a primer-determined specificity of 100% [62, 63]. In contrast, sensitivity for the combined microscopy technique with a single stool sample ranges between 86 and 95% for nematodes [61].

In Colombia, studies have until now addressed intestinal parasitism in children and the general population with prevalence ranges reported between 11 and 50% [2, 21]. The overall 41% prevalence of IPI in pregnant women reported here is higher than that reported in similar studies in Mexico [69], Brazil [34], northwestern and southern Ethiopia [70, 71], with prevalence figures of 38%, 33%, 32%, and 19% respectively. However, it is lower than the 74% and 81% prevalence found in studies in pregnant women in Venezuela [19] and New Guinea [72].

We found a low prevalence of pathogenic intestinal parasites including *Giardia lamblia* and *Ascaris lumbricoides*. These results contrasted sharply with similar studies reporting higher prevalence. For *G. lamblia*, the 1% prevalence reported here was lower than the reported range of 3–66% in a Brazilian cohort [34], two African studies [70, 71] and Mexico [69]. Finally, for *A. lumbricoides*, the only helminth found in this study, the prevalence of 0.3% reported here contrasted with the range of 2.9–28% from similar studies in Ethiopia [70], Mexico [69], and Ecuador [73]. For *E. histolytica/dispar* complex, the 1.5% prevalence reported here was lower than the 8% and 12% prevalence found in studies from Venezuela [19] and Ethiopia [70].

In this study, most positive findings were non-pathogenic intestinal parasites. *Blastocystis hominis* appeared in 25% of stool samples with the combined microscopy technique. This protozoon is most frequently identified in fecal samples worldwide [38, 74, 75], with reported prevalences above 50% [37] and as high as 100% in developing countries [38]. Colombian studies in school children identified this parasite as common, with over 50% prevalence [21, 39]. Despite its debated pathogenicity, *Blastocystis hominis* has been reported to cause or be associated with abdominal pain and diarrhea [76], hematological abnormalities [77], pregnancy-related anemia [78], and immunosuppression [79]. Additionally, Ramirez et al. [80] reported that 72% of children positive for *Blastocystis hominis* were asymptomatic, with just 11% showing abdominal pain and 2% diarrhea. In contrast, a study of outpatients with digestive disorders in Spain reported a 7% prevalence of *Blastocystis hominis* infestation [81]. The major routes of transmission include drinking water, food, direct human-to-human contact, and zoonotic infections [76]. Aside from *Blastocystis hominis*, other prevalent non-pathogenic parasites included *Endolimax nana* and *Entamoeba coli*. Regarding *Endolimax nana*, in this study, the prevalence of 15% in pregnant women contrasts with the 2–4% in other South American studies [23, 34]. For *Entamoeba coli*, the prevalence of 5% in this study is lower than the reported range between 6 and 19% in similar cohorts [23, 34] and the 9% reported in HIV-infected Tanzanian women [82].

The overall prevalence of non-pathogenic and pathogenic species can be interpreted as an important semi-quantitative indicator of the intensity of fecal–oral routes of transmission and/or contamination of food and water with feces within the study areas [21]. In this study, the dissimilar prevalence of pathogenic and non-pathogenic intestinal parasites may be explained by separately exploring the host, environment, and parasitic factors. The hosts, in this study, are pregnant women. Overall, intestinal parasites are more frequent in children and young adults than in older adults [83], facilitated, among other things, by limited hygiene habits and frequent consumption of contaminated water in these age groups [84]. The good quality of water for human consumption in Bogotá, relative to other geographical areas in Colombia, corresponds to a low risk of waterborne morbidity and mortality [85]. Open availability of broad-spectrum antiparasitic drugs [84] may occur through public health campaigns and higher access of vulnerable populations to primary care units in Bogotá. Better educational level and appropriate sanitary facilities play an important role in the prevention of parasitic infections [86], as is the case in Bogotá. Finally, the average temperature of 14 °C in Bogotá does not favor most helminthic life cycles. However, commensal non-pathogenic protozoal species are less demanding in terms of environmental conditions [21]. It is possible that these commensal parasites have more permissive growth requirements in pregnant women compared with pathogenic parasites.

In this study, any intestinal parasite could be detected in 41% of pregnant women. This is comparable to similar studies in Ethiopia and Brazil [16, 34, 70], reporting prevalence between 32 and 57%. Given that many of the IPI share common routes of transmission, polyparasitism is a common occurrence in exposed populations. In this research, the prevalence of polyparasitism was 9%, within the range found in similar studies which reported prevalence as low as 6.6% in Ethiopian pregnant women [16] and up to 33% in a Brazilian cohort [34]. This variability may be explained by geographic differences, age variations, diversity of health conditions and cultural practices in different study areas [70].

For pregnant women infected with any intestinal parasite, two risk factors appeared to be important, namely the time since the last deworming procedure and civil status. Pregnant women who had never been dewormed showed a statistically significant higher prevalence of infection by any intestinal parasite. However, although prophylactic deworming programs in children and women of reproductive age have been recommended [87, 88], their effectiveness in health outcomes is unclear [89, 90]. Our finding may support the effectiveness of deworming programs as we could show a decreasing prevalence of intestinal parasites. Women who were married or living with a partner showed a non-significant trend toward a higher prevalence of intestinal parasitism. Derso et al. [70] did not find a difference in IPI in pregnant women when considering marital status. In contrast, van Eijk et al. [13] found that married women had a lower prevalence of hookworm infections.

The prevalence of polyparasitism was higher in women from minority groups. These groups, which include indigenous people, African Colombians, and Raizals (from the Caribbean island), likely immigrated into Bogotá from other regions with a high risk of polyparasitism [21] and may have arrived with undiagnosed infestations. Prevalence of polyparasitism was also higher in pregnant women living without a water sink. Having a water sink facilitates the hygienic habit of washing hands before and after going to the bathroom and after changing diapers. Although this variable was assessed recently in Kenyan children living in urban slums [91], it has not been specifically assessed in studies with pregnant women.

## Conclusions

This study has been the first conducted with pregnant women in Colombia that estimates the prevalence of and factors associated with intestinal parasitism, evaluating vulnerable populations living in conditions of poverty and social inequality in the largest city in the country. A low prevalence of pathogenic parasites was found in pregnant women. At the same time, a high prevalence of parasites was identified with disputed pathogenicity indicating fecal–oral contamination. A significant association was identified between time since last deworming and infection by any parasite. Higher but non-significant prevalence of intestinal parasites was found in pregnant women living with a partner (by any parasite infection) and in pregnant women belonging to minority ethnic groups and those without handwashing facilities (by polyparasitism).

This study provides guidance to health authorities regarding some risk factors to prevent intestinal parasitism in pregnant women. The high prevalence of parasites with debated pathogenicity points to maintaining and strengthening educational interventions to eliminate fecal–oral transmission routes, including handwashing during daily activities, particularly after using washrooms and, in child caring settings, after diaper changes and cleaning. Owing to their harmful potential, it is important to continue research that identifies causes for the high prevalence of commensal intestinal parasites.

## Abbreviations

CDC: Center for Disease Control and Prevention
CI: Confidence interval
Ct: Cycle threshold value
DHS: Demographic and Health Survey
DNA: Deoxyribonucleic acid
HIV: Human immunodeficiency virus
IPI: Intestinal parasitic infections
OR: Odds ratio
PCR: Polymerase chain reaction
qPCR: Quantitative polymerase chain reaction
STH: Soil-transmitted helminthiasis
UPA: Unidad Primaria de Atención; Primary Attention Unit
USD: United States dollar
WB: WB kit (sodium iodide method)
WHO: World Health Organization
